# Famitinib in combination with concurrent chemoradiotherapy in patients with locoregionally advanced nasopharyngeal carcinoma: a phase 1, open-label, dose-escalation Study

**DOI:** 10.1186/s40880-018-0330-z

**Published:** 2018-11-01

**Authors:** Qiuyan Chen, Linquan Tang, Na Liu, Feng Han, Ling Guo, Shanshan Guo, Jianwei Wang, Huai Liu, Yanfang Ye, Lu Zhang, Liting Liu, Pan Wang, Yingqin Li, Qingmei He, Xiaoqun Yang, Qingnan Tang, Yang Li, YuJing Liang, XueSong Sun, Chuanmiao Xie, Yunxian Mo, Ying Guo, Rui Sun, Haoyuan Mo, Kajia Cao, Xiang Guo, Musheng Zeng, Haiqiang Mai, Jun Ma

**Affiliations:** 1 State Key Laboratory of Oncology in South China, Collaborative Innovation Center for Cancer Medicine, Guangdong Key Laboratory of Nasopharyngeal Carcinoma Diagnosis and Therapy, Sun Yat-sen University Cancer Center, Guangzhou, 510060 P. R. China; 2 Department of Nasopharyngeal Carcinoma, Sun Yat-sen University Cancer Center, Guangzhou, 510060 P. R. China; 3 Department of Ultrasound, Sun Yat-sen University Cancer Center, Guangzhou, 510060 P. R. China; 4 Department of Radiation Oncology, Key Laboratory of Translational Radiation Oncology, Hunan Cancer Hospital and The Affiliated Cancer Hospital of Xiangya School of Medicine, Central South University, Changsha, 410013 P. R. China; 5 Department of Science and Education, Sun Yat-sen Memorial Hospital, Guangzhou, 510120 P. R. China; 6 Department of Radiation Oncology, The First Affiliated Hospital of Guangzhou Medical University, Guangzhou, 510120 P. R. China; 7 Department of Imaging, Sun Yat-sen University Cancer Center, Guangzhou, 510060 P. R. China; 8 Department of Clinical Trial Center, Sun Yat-sen University Cancer Center, Guangzhou, 510060 P. R. China; 9 Department of Radiation Oncology, Sun Yat-sen University Cancer Center, Guangzhou, 510060 P. R. China

**Keywords:** Nasopharyngeal carcinoma, Famitinib, Concurrent chemoradiotherapy, Phase I, dynamic contrast-enhanced ultrasound

## Abstract

**Background:**

Famitinib is a tyrosine kinase inhibitor against multiple targets, including vascular endothelial growth factor receptor 2/3, platelet-derived growth factor receptor, and stem cell factor receptor (c-kit). Previous studies have demonstrated anti-tumour activities of famitinib against a wide variety of advanced-stage solid cancers. We aimed to determine the safety and efficacy of famitinib with concurrent chemoradiotherapy (CCRT) in patients with locoregionally advanced nasopharyngeal carcinoma (NPC). We also evaluated the feasibility of contrast-enhanced ultrasound (D-CEUS) as a predictor of early tumour response to famitinib and to correlate functional parameters with clinical efficacy.

**Methods:**

The trial was conducted in subjects with stage III or IVa-b NPC using a 3 + 3 design of escalating famitinib doses. Briefly, subjects received 2 weeks of famitinib monotherapy followed by 7 weeks of famitinib plus CCRT. D-CEUS of the neck lymph nodes was performed at day 0, 8 and 15 after famitinib was administered before starting concurrent chemoradiotherapy. End points included safety, tolerability and anti-tumour activity.

**Results:**

Twenty patients were enrolled (six each for 12.5, 16.5 and 20 mg and two for 25 mg). Two patients in the 25 mg cohort developed dose-limiting toxicities, including grade 4 thrombocytopenia and grade 3 hypertension. The most common grade 3/4 adverse events were leukopenia, neutropenia and radiation mucositis. D-CEUS tests showed that more than 60% of patients achieved a perfusion parameter response after 2 weeks taking famitinib alone, and the parameter response was associated with disease improvement. In the famitinib monotherapy stage, three patients (15%) showed partial responses. The complete response rate was 65% at the completion of treatment and 95% 3 months after the treatment ended. After a median follow-up of 44 months, the 3-year progression-free survival (PFS) and distant metastasis-free survival were 70% and 75%, respectively. Subjects with a decrease of perfusion parameter response, such as peak intensity decreased at least 30% after 1 week of famitinib treatment, had higher 3-year PFS (90.9% vs. 44.4%, 95% CI 73.7%–100% vs. 11.9%–76.9%, *P *< 0.001) than those with an increase or a reduction of less than 30%.

**Conclusions:**

The recommended famitinib dose for phase II trial is 20 mg with CCRT for patients with local advanced NPC. D-CEUS is a reliable and early measure of efficacy for famitinib therapies. Further investigation is required to confirm the effects of famitinib plus chemoradiotherapy.

**Electronic supplementary material:**

The online version of this article (10.1186/s40880-018-0330-z) contains supplementary material, which is available to authorized users.

## Background

Nasopharyngeal carcinoma (NPC) is highly endemic in Southern China and Southeast Asia, with a peak incidence of 50 cases per 100,000 [1]. Concurrent chemoradiotherapy (CCRT) with or without adjuvant chemotherapy is currently considered as the standard therapeutic regimen for locoregionally advanced NPC [2–6]. A previous study from this research group [7] demonstrated that induction chemotherapy with cisplatin, fluorouracil, and docetaxel in addition to CCRT significantly improved survival versus CCRT alone for advanced NPC patients. However, the role of induction chemotherapy remains debatable. Currently, experts agree that concurrent use of cisplatin with radiation improves progression-free survival (PFS) and overall survival (OS) [6, 8–11].

Intensity-modulated radiotherapy (IMRT) could target irregularly shaped tumour in a region surrounded by multiple critical organs, and has been increasingly used [12–15]. Irrespective of the availability of modern treatments, up to 30% of patients with locoregionally advanced NPC still die of distant metastasis [14].

Angiogenesis is essential for tumour growth and metastasis, and vascular endothelial growth factor (VEGF) is one of the most studied angiogenic factors. VEGF expression is associated with metastasis in NPC patients [16, 17]. Anti-VEGF antibody bevacizumab has direct anti-vascular effects with enhanced radiosensitivity [18]. A phase II study showed that the addition of bevacizumab to standard chemoradiation treatment in NPC patients is feasible and could delay the progression of subclinical distant disease [19].

Receptor tyrosine kinase (RTK) inhibitors with multiple targets are also promising for NPC treatment. Expression of the c-kit and platelet-derived growth factor receptor (PDGFR) has been detected in NPC tissues, cell lines and tumour xenografts [20–24]. In preclinical models, RTK inhibitors, such as sunitinib, demonstrated encouraging results in NPC [25, 26]. A phase II trial demonstrated significant responses in recurrent or metastatic NPC patients treated with sunitinib [27]. However, the trial was terminated due to haemorrhagic events occurred in 69% of the patients [27]. Therefore, new multi-target RTK inhibitors with acceptable safety profiles are needed for NPC.

The sunitinib analogue famitinib is a novel and highly potent multi-target RTK inhibitor against VEGFR, C-Kit, and PDGFR, and has anti-tumour activity in a range of solid tumours [28–30]. The pharmacokinetic data showed that the mean half-lives and major metabolite of famitinib in healthy volunteers were shorter than those of sunitinib [28, 30, 31]. Furthermore, after administration for 28 days, the degrees of famitinib accumulation in vivo were significantly lower than sunitinib [28, 30, 31], indicating that famitinib may be a safer agent. Preclinical studies have demonstrated that both famitinib and sunitinib are synergistic with radiation [32, 33]. On the basis of promising preclinical data, we conducted this phase I study to evaluate the safety, tolerability and dose-limiting toxicities (DLTs) of famitinib with CCRT in NPC patients. The secondary objectives were to assess the anti-tumour activity of famitinib. Previous study has demonstrated that using contrast-enhanced ultrasound (D-CEUS) as a tool to predict early treatment response for metastatic renal cell carcinoma treated with sunitinib. We also evaluate whether D-CEUS could be used to predict famitinib response.

## Patients and methods

### Patients

This open-label, dose-escalation phase I study enrolled treatment-naïve patients with pathologically proven locoregionally advanced NPC who sought treatment between November 11, 2011 and September 23, 2013 at Sun Yat-sen University Cancer Center, Guangzhou, China. NPC was staged according to the 7th edition American Joint Committee on Cancer [AJCC] staging system. Patients with histologically confirmed undifferentiated NPC, WHO III and confirmed T3-4N1M0 or T1-4N2-3M0 locoregionally advanced NPC were eligible. Other inclusion and exclusion criteria are described in detail in Additional file 1: Methods.

The study protocol was approved by the Ethics Committee of Sun Yat-sen University Cancer Center (Approval No.: A2011-021-01). All patients provided written informed consent to the study. The study was registered at https://register.clinicaltrials.gov (NCT01462474).

### Study design and procedures

This trial used a standard 3 + 3 design to identify the maximum tolerated dose. Sequential dose-escalation cohorts of three to six patients were given oral famitinib at a starting dose of 12.5 mg/day, which was increased to 16.5, 20, and 25 mg/day. Famitinib alone was administered for 2 weeks prior to starting chemoradiotherapy, followed by 7 weeks of famitinib plus CCRT. Dose escalation was continued until DLTs or until the highest planned dose level without any DLT. If one out of three patients had a DLT, three additional patients were added at that dose. If two out of six patients had a DLT, the dose was declared to be above the maximum tolerated dose.

IMRT was conducted as previously reported [7]. Gross tumour volume included the primary tumour and the enlarged lymph nodes. The definition of planning target volumes (PTVs), high- (CTV-1) and low-risk clinical target volume (CTV-2) are detailed in Additional file 1: Methods.

Cisplatin was administered at 100 mg/m^2^ on day 1, 22, and 43 of radiotherapy. Cisplatin dose reductions or delays were based on a predefined toxicity criterion, which is available in Additional file 1: Methods. Considering the maximum tolerated dose of famitinib was 25 mg for advanced solid malignancy [28], we chose an initial dose of 12.5 mg. If two out of three patients had a DLT at 12.5 mg, the concurrent cisplatin dose was reduced to 80 mg/m^2^ for the remaining patients.

### Assessments

Toxicities were assessed by the National Cancer Institute Common Terminology Criteria for Adverse Events (CTCAE, version 4.0). DLTs included grade 4 thrombocytopenia (or grade 3 with haemorrhage), grade 4 neutropenia (< 1.0 × 10^9^/L) lasting for more than 5 days (or grade 3 with fever at > 38.5 °C), grade 4 anaemia, and any other grade 3 non-hematologic toxicity. Tumour response [i.e., complete response (CR), partial response (PR), progressive disease (PD) and stable disease (SD)] was evaluated 2 weeks after taking famitinib, at completion of treatment and 12 weeks later according to Response Evaluation Criteria in Solid Tumours (RECIST version 1.1).

### Contrast-enhanced ultrasound (D-CEUS)

Recent evidence has suggested that molecular anti-angiogenic agents often induce tumour necrosis or decrease tumour vascularity before a reduction in tumour volume [34–36]. Therefore, D-CEUS of the neck lymph nodes was performed at baseline (day 0), day 8 and 15 after famitinib was administered before starting CCRT. The ultrasonography protocol, enhancer agent SonoVue (Bracco, Milan, Italy), and quantitative analysis of D-CEUS data are described in detail in Additional file 1: Methods [37, 38]. Six perfusion parameters sufficient to characterize both blood volume and blood flow were extracted from time-intensity curves: peak intensity (PI), area under the curve (AUC), time to PI (TP), mean transit time (MTT), slope of wash-in (PW) and wash-in perfusion index (WIPI). The above parameters are defined in Additional file 1: Methods. Intra-observer variability and inter-observer variability between two operators (FH and JWW) was calculated for the entire D-CEUS process (D-CEUS examination, ROI drawing and calculation of perfusion parameters) by evaluating 3 repeated examinations (SonoVue bolus injection repeated every 15 min) on 10 different patients.

### Immunohistochemistry and quantitative PCR

Tissues were biopsied and routinely paraffin-embedded. VEGFR2, PDGFR2 and C-Kit expression was examined by immunohistochemistry as detailed in Additional file 1: Methods. Furthermore, blood samples were collected to determine plasma VEGF and PDGF and stem cell factor (SCF) levels at day 0 and 15 after famitinib therapy and 12 weeks after completing CCRT (Additional file 1: Methods). Plasma EBV DNA concentrations were routinely measured by quantitative PCR as we described previously [39, 40].

### End points

The primary end points were safety of famitinib combined with CCRT. The secondary end point was tumour response. We also evaluated whether the functional parameters of D-CEUS could serve as effective predictors of early tumour response to famitinib and the correlation between the functional parameters and clinical efficacy. Follow-up assessments were performed every 3 months during the first 2 years, every 6 months during years 3–5, and then every year.

### Statistical analysis

Non- normally distributed continuous variables were expressed as median (IQR) and normally distributed data were expressed as mean (SD). Categorical variables were presented as number and percentage (%). Progression-free survival (PFS) was calculated from the date of entry into the trial to the date of first failure (local and/or regional persistence/recurrence or distant metastasis) or death from any cause or the date of the last follow-up. Distant metastasis-free survival (DMFS) was calculated from the date of entry into the trial to the date of distant relapse or death from any cause or the date of the last follow-up. Survival analyses were performed by the Kaplan–Meier method, and log-rank test was used to compare two groups of patients with decreased D-CEUS functional parameters (≥ 30% vs. < 30%). The percent coefficient of change (CV, calculated by dividing the SD by the mean and multiplying by 100) for the perfusion parameters was calculated to evaluate intra-observer variability, and the intra-class correlation coefficients for the six perfusion parameters were also estimated to evaluate inter-observer variability. All statistical analyses were performed using IBM SPSS version 20.0.

## Results

### Patient demographic and baseline characteristics

**Fig. 1:**
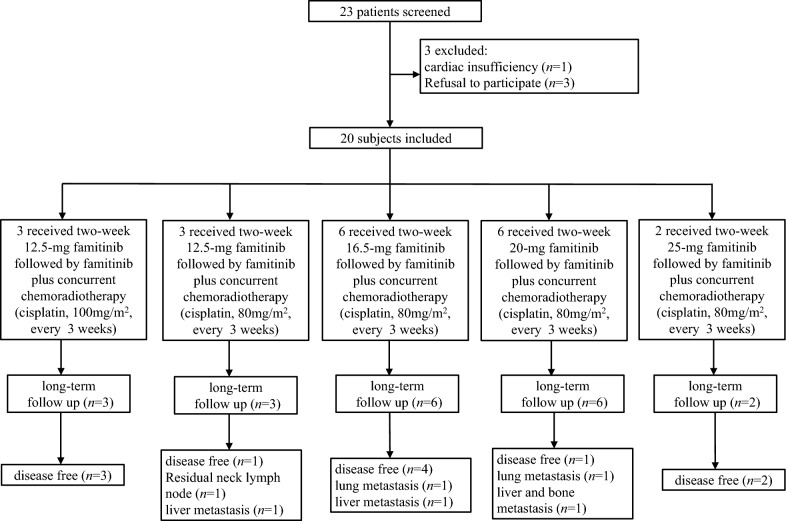
Flow chart of patients enrolled in this clinical trial

The study flowchart is shown in Fig. 1. Twenty-three patients were screened for eligibility. One patient was excluded due to cardiac insufficiency and two patients were not included because they refused to provide consent. Finally, 20 patients were enrolled in the study. The median age of the patients was 43 years (range 26–56 years) and 80% (18/20) of the patients were male. Patient demographic and baseline characteristics are shown in Table 1. Three patients received 2 weeks of famitinib (12.5 mg/day) followed by famitinib plus CCRT (cisplatin, 100 mg/m^2^). Because two of the three patients receiving CCRT plus 12.5 mg cisplatin at the initial dose had a DLT, cisplatin was reduced to 80 mg/m^2^ in the remaining patients. Finally, three, six, six and two patients were included in the 12.5, 16.5, 20 and 25 mg cohorts.

**Table 1:** Demographic and baseline characteristics of patients with NPC who were treated with famitinib

Variables	Patients (n = 20)
Age, years	
Median (IQR)	43 (39–48)
Range	26–56
Male sex	18 (80%)
ECOG	
0	1 (5%)
1	19 (95%)
Histology, WHO type III	20 (100%)
Tumour stage	
T1	1 (5%)
T2	3 (15%)
T3	13 (65%)
T4	3 (15%)
Node stage	
N1	2 (10%)
N2	13 (65%)
N3	5 (25%)
Clinical stage	
III	12 (60%)
IVa	3 (15%)
IVb	5 (25%)
EBV DNA,	
≥ 4000 copy/ml	10 (50%)
VCA-IgA	
≥ 1:80	15 (75%)
EA-IgA	
≥ 1:10	13 (65%)
Smoking^a^	
Yes	9 (45%)
Family history of NPC	
Yes	3 (15%)

*ECOG* Eastern Cooperative Oncology Group, *WHO* World Health Organization, *EBV DNA* Epstein–Barr virus DNA, *VCA* viral capsid antigen, *IgA* immunoglobulin A, *EA*, early antigen, *NPC* nasopharyngeal carcinoma

^a^Defined as smoking ≥100 cigarettes/lifetime

### Co-primary end points

Neither radiotherapy interruptions nor deaths occurred during the study. Famitinib as a single agent was generally well tolerated. Except one patient with grade 3 adverse event (hematuria), all adverse events were grade 1 or 2 (Table 2).

**Table 2:** Treatment-emergent adverse events occurring in NPC patients during the study in the safety analysis set

Adverse event	Famitinib alone					Famitinib with CCRT				
	Grade 1–2	Grade 3	Grade 4	Grade 5	Total	Grade 1–2	Grade 3	Grade 4	Grade 5	Total
Leukopenia	1 (5%)	0	0	0	1 (5%)	2 (10%)	17 (85%)	1 (5%)	0	20 (100%)
Neutropenia	1 (5%)	0	0	0	1 (5%)	9 (45%)	10 (50%)	1 (5%)	0	20 (100%)
Anaemia	2 (10%)	0	0	0	2 (10%)	16 (80%)	4 (20%)	0	0	20 (100%)
Radiation mucositis	0	0	0	0	0	16 (80%)	3 (15%)	1 (5%)	0	20 (100%)
Nausea and vomiting	0	0	0	0	0	16 (80%)	1 (5%)	0	0	17 (85%)
Radiation dermatitis	0	0	0	0	0	13 (65%)	0	0	0	13 (65%)
Weight loss	0	0	0	0	0	15 (75%)	0	0	0	15 (75%)
Proteinuria	1 (5%)	0	0	0	1 (5%)	15 (75%)	1 (5%)	0	0	16 (80%)
Thrombopenia	0	0	0	0	0	10 (50%)	3 (15%)	1 (5%)	0	14 (70%)
Hypertension	3 (15%)	0	0	0	3 (15%)	8 (40%)	1 (5%)	0	0	9 (45%)
Liver function impairment	3 (15%)	0	0	0	3 (15%)	10 (50%)	1 (5%)	0	0	11 (55%)
Hypertriglyceridemia	5 (25%)	0	0	0	5 (25%)	5 (25%)	0	0	0	5 (25%)
Hearing impairment	0	0	0	0	0	4 (20%)	0	0	0	4 (20%)
Renal impairment	0	0	0	0	0	4 (20%)	0	0	0	4 (20%)
Hematuria	2 (10%)	1 (5%)	0	0	3 (15%)	4 (20%)	1 (5%)	0	0	5 (25%)
Haemorrhage	0	0	0	0	0	2 (10%)	0	0	0	2 (10%)
Skin rash	2 (10%)	0	0	0	2 (10%)	1 (5%)	0	0	0	1 (5%)
Hypothyroidism	0	0	0	0	0	0	0	0	0	0
Hypercholesterolemia	1 (5%)	0	0	0	1 (5%)	0	0	0	0	0
Elevated total bilirubin	1 (5%)	0	0	0	1 (5%)	1 (5%)	0	0	0	1 (5%)
Elevated GGT	2 (10%)	0	0	0	2 (10%)	2 (10%)	0	0	0	2 (10%)

*CCRT* concurrent chemoradiotherapy, *GGT* gamma glutamyl transpeptidase, *NPC* nasopharyngeal carcinoma

More adverse events were observed with famitinib plus CCRT (Table 2). The majority of adverse events were grade 1 or 2. The five most frequent adverse events were leukopenia (100%), neutropenia (100%), anemia (100%), radiation mucositis (100%), and nausea and vomiting (85%). The five most frequent grade 3 or 4 adverse events were leukopenia (90%), neutropenia (55%), thrombopenia (20%), anaemia (20%) and radiation mucositis (20%). In addition, grade 1 or 2 hemorrhage occurred in 2 (10%) patients and no grade 3–4 haemorrhage was recorded. No grade 5 adverse event was reported. Two out of three patients receiving 12.5 mg cisplatin had DLTs; one patient suffered grade 4 neutropenia lasting more than 5 days, and the other patient suffered grade 3 neutropenia with fever. After cisplatin was reduced to 80 mg/m^2^, in the remaining patients, one patient had grade 4 thrombocytopenia and one patient had grade 3 hypertension while receiving 25 mg cisplatin. Grade 3 hearing impairment occurred in one patient; no other grade 3 or 4 late adverse events were reported (Additional file 1: Table S1).

Fifteen (15/20, 75.0%) patients completed three cycles and 5 (25.0%) completed two cycles of cisplatin. The recommended phase II dose was defined as famitinib 20 mg/day with CCRT. Detailed treatments are presented in Additional file 1: Table S2.

### Secondary end point

**Fig. 2:**
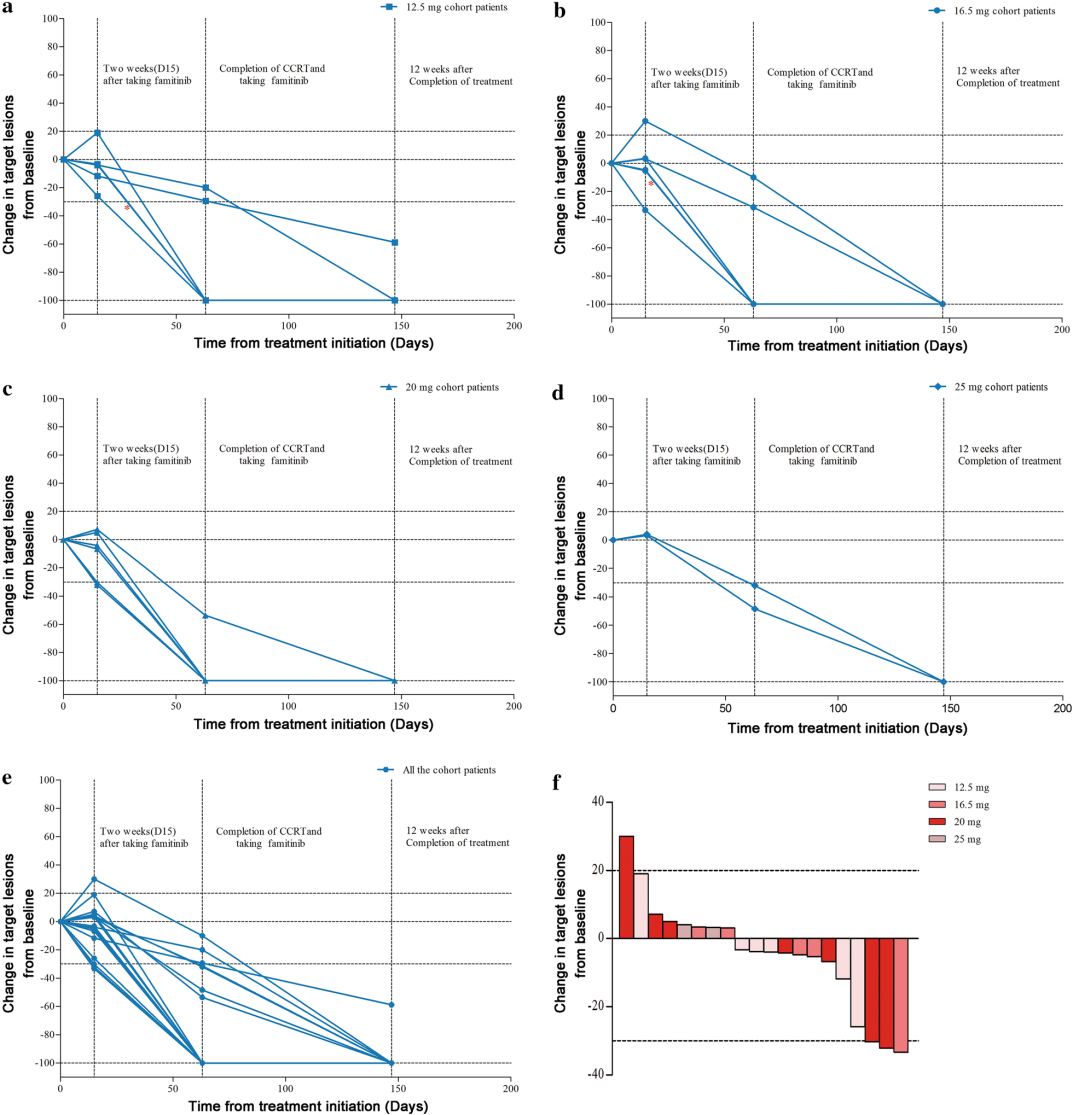
Characteristics of neck lymph node regression in patients with nasopharyngeal carcinoma receiving famitinib and concurrent chemoradiotherapy. The response was measured as the largest percentage reduction in the sum of the longest diameters of target lesions for all assessable patients with a radiographic assessment (n = 20). Response kinetics in patients receiving famitinib **a** 12.5 or **b** 16.5 or **c** 20 or **d** 25 mg cohort and all the patients (**e**). Tumours were assessed 2 weeks after taking famitinib (D15), at the end of CCRT and 12 weeks after treatment according to the RECIST (version 1.0) guidelines; horizontal line at − 30% marks the threshold for defining objective response (partial tumour regression) according to RECIST, and a horizontal line at − 20% indicates the threshold for defining progressive disease. **f** Waterfall plot of best tumour response 2 weeks after taking famitinib (D15). *Indicated that two lines overlapped together

In famitinib monotherapy, 3 (15%) patients exhibited PR, 1 (5%) patient had PD, and 16 (80%) patients had SD (Fig. 2a–e). Overall, 12 (60%) patients demonstrated tumour shrinkage (from − 3.3% to− 33.3%, Fig. 2e, f).

Thirteen (65.0%) patients achieved CR and seven (35.0%) patients achieved PR at the completion of CCRT, and 19 (95%) patients achieved CR at 3 months after treatment (Fig. 2e). One patient, who had a residual neck lymph node that was evaluated 9 months after CCRT, subsequently underwent selective neck dissection. Five patients developed distant organ metastasis during 3 years of follow-up. After a median follow-up of 44 months, the 1-, 2-, and 3-year PFS was 85%, 70% and 70%, respectively. Additionally, the 1-, 2-, and 3-year DMFS was 90%, 75% and 75%, respectively (Additional file 1: Fig. S4). All five metastatic patients received palliative chemotherapy, and three patients were currently alive and two patients died.

### Early Perfusion parameters response associated with clinical outcome

The mean CV was 4.05%, 6.63%, 3.64%, 24.40%, 6.44% and 6.33% for PI, AUC, TP, MTT, PW and WIPI, respectively. The intra-class correlation coefficients for the six perfusion parameters were between 0.95 and 0.99, indicating good agreement between observers.

**Fig. 3:**
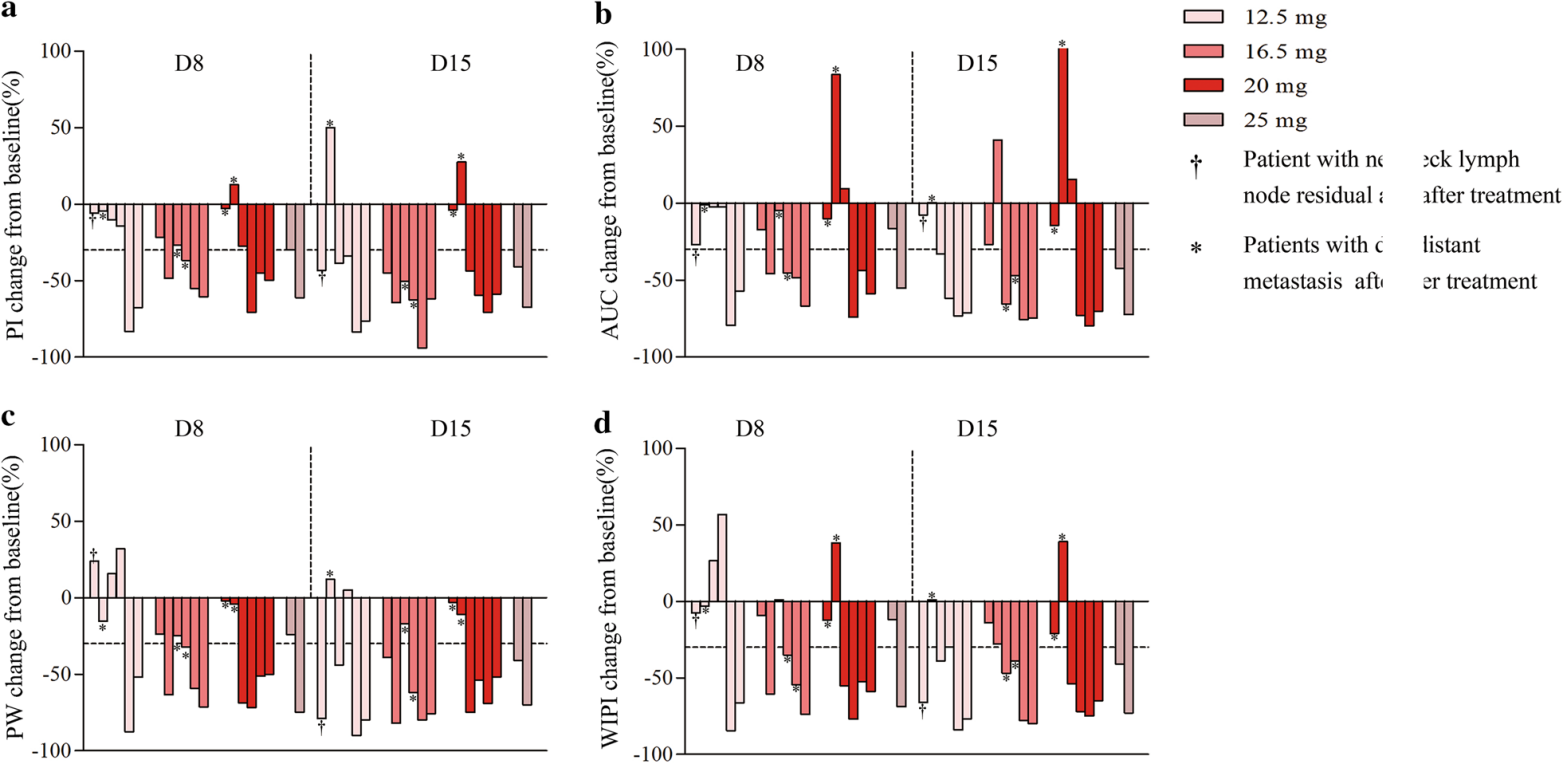
Percentage change in functional parameters of D-CEUS on day 8 and 15 from baseline. **a** *PI* peak intensity, **b** *AUC* area under the curve, **c** *PW* slope of wash-in, **d** *WIPI* wash-in perfusion index. Data truncated at 100%. Horizontal line at − 30% marks threshold for functional parameters response

**Fig. 4:**
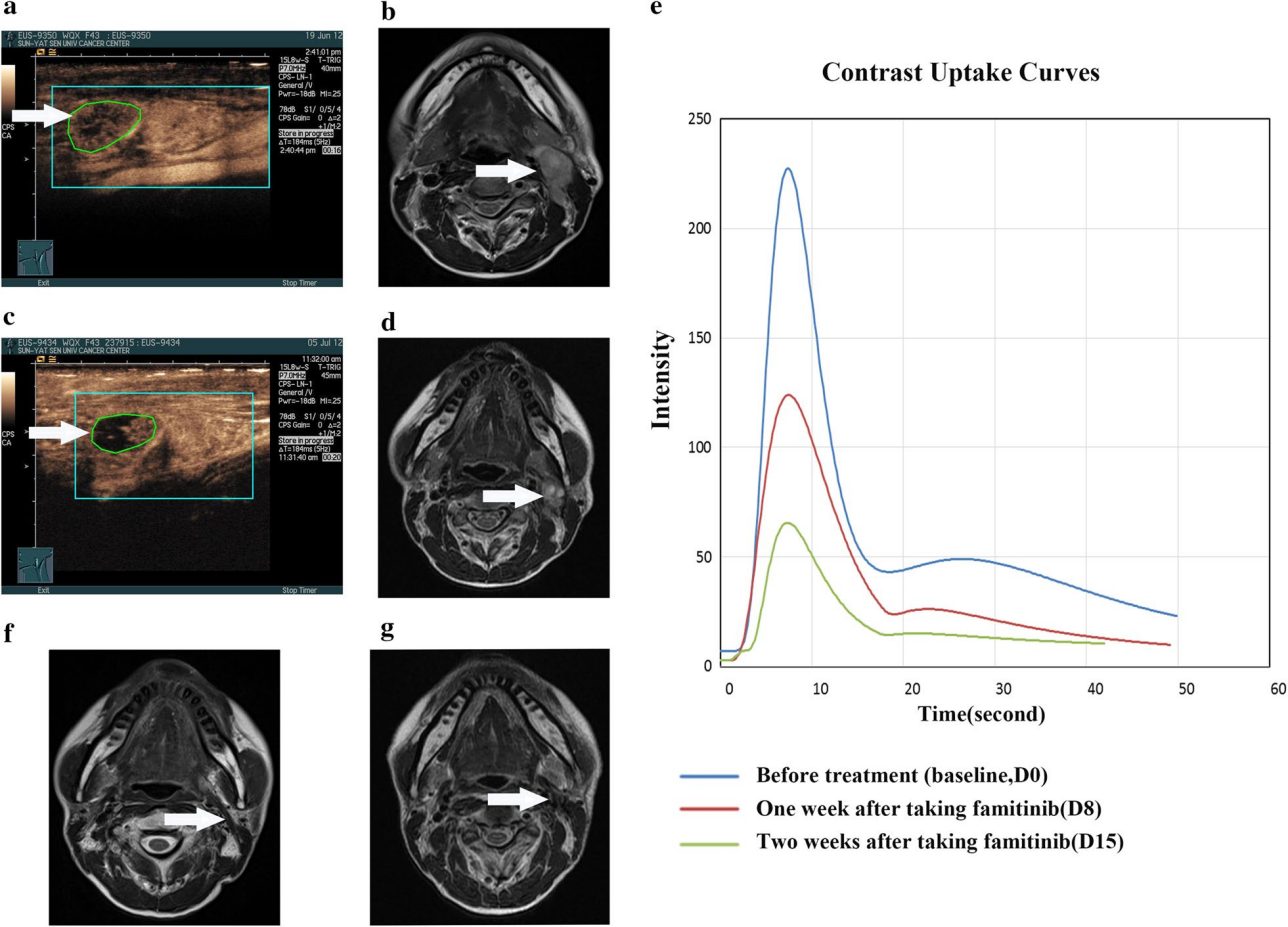
Target neck lymph node lesion in a 43-year-old woman (staged with T2N2M0) treated with famitinib (20 mg) and CCRT: clinical example of the partial response (PR) according to RECIST (version 1.1.). **a** Contrast-enhanced ultrasound with a strong vascularized lesion (arrow) and corresponding time intensity curve at baseline. **b** The metastatic neck lymph node lesion was evident in the axial T2-weighted MRI image at baseline (arrow). **c** Fourteen days after the onset of famitinib alone treatment, D-CEUS revealed an increase in tumour necrosis with a drastic reduction of the tumour perfusion parameters, as shown by the contrast enhancement pattern and corresponding time-intensity curve. **d** The longest diameter of the metastatic neck lymph node lesion greatly regressed in the axial T2-weighted MRI image at D15 (arrow). **e** Time-intensity curves of tumour enhancement at baseline (blue curve), on D8 (red curve) and on D15 (green curve). It was possible to observe a reduced maximum enhancement and lower area under the enhancement curve early after treatment. **f**, **g** The metastatic neck lymph node lesion disappeared after the completion of CCRT and famitinib treatment (arrow) and conformed 3 months later (arrow). The patient was disease-free after long-term follow-up

Anti-angiogenic activity was noted across all four doses. At baseline, the frequency of VEGFR2-positive tumour cells was 50% or higher in 16 (80%) patients and that of C-kit-positive tumour cells was 50% or higher in 8 (40%) patients. PDGFR expression was not detected in NPC tissues (Additional file 1: Fig. S1). The plasma VEGF and PDGF levels decreased versus baseline after 2 weeks of single famitinib therapy and slightly increased after discontinuing famitinib 3 months later (Additional file 1: Fig. S2). Furthermore, 11 (55%), 10 (50%), 11 (55%), and 11 (55%) patients exhibited an at least 30% reduction in perfusion parameter response 1 week after taking famitinib for PI, AUC, PW, and WIPI, respectively. Seventeen (85%), 13 (65%), 17 (85%), and 11 (65%) patients exhibited response at 2 weeks, respectively (Fig. 3). There was no statistically significant difference in changes in the perfusion parameters at baseline, day 8 and 15 in terms of tumour response (PR vs SD/PD) after taking famitinib for 2 weeks (data not shown). However, tumour necrosis of neck lymph nodes was observed on day 15 in several typical cases (Fig. 4).

**Fig. 5:**
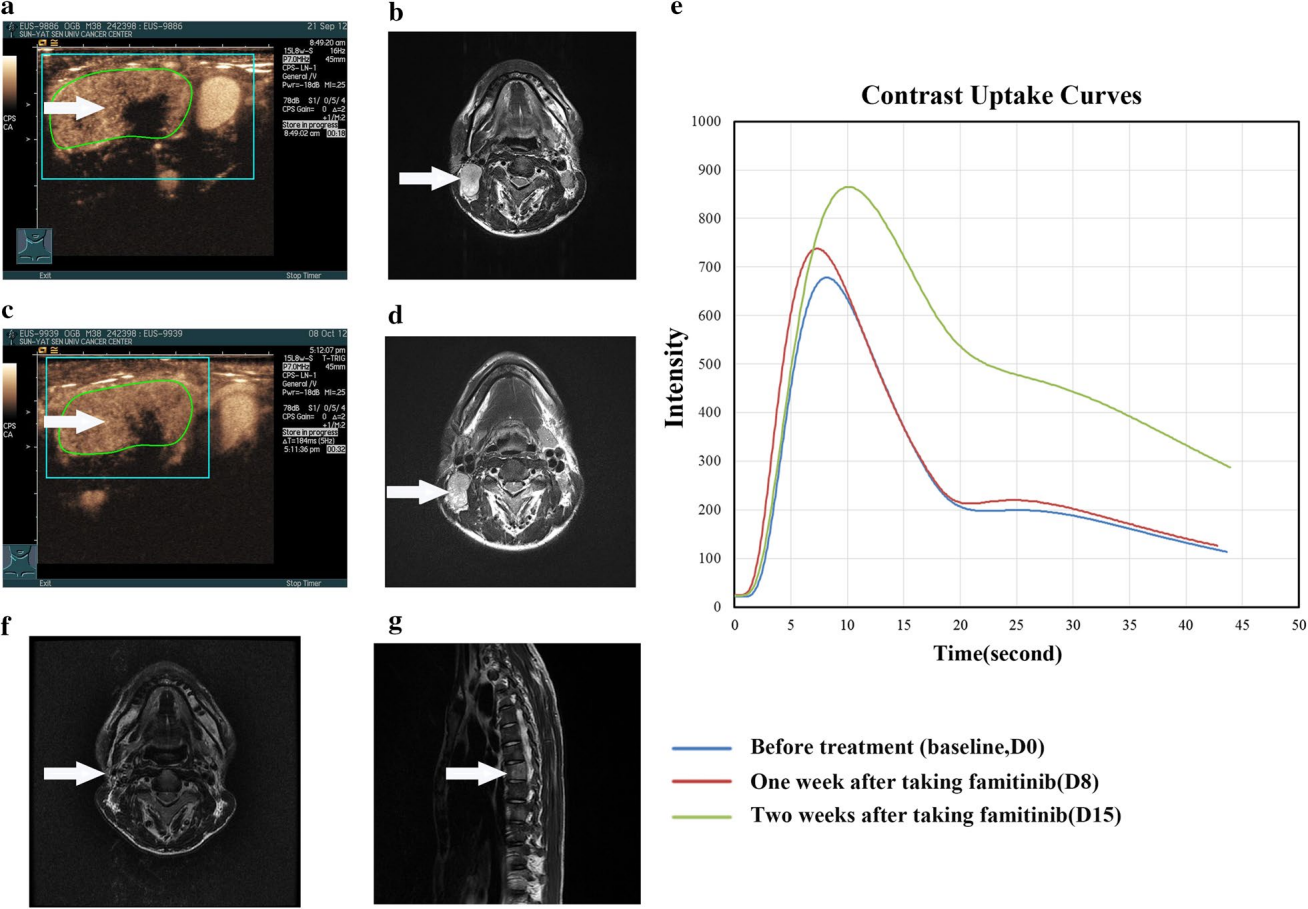
Target neck lymph node lesion in a 39-year-old man (staged with T4N2M0) treated with famitinib (20 mg) and CCRT: clinical example of stable disease (SD) according to RECIST (version 1.1.). **a** Contrast-enhanced ultrasound with a strong vascularized lesion (arrow) and corresponding time intensity curve at baseline. **b** The metastatic neck lymph node lesion was evident in the axial T2-weighted MRI image at baseline (arrow). **c** Fourteen days after the onset of famitinib alone treatment, D-CEUS revealed an enhancement in tumour vascularity density with a drastic increase of tumour perfusion parameters, as shown by the contrast enhancement pattern and corresponding time-intensity curve. **d** The longest diameter of the metastatic neck lymph node lesion did not change in the axial T2-weighted MRI image at D15 (arrow). **e** Time-intensity curves of tumour enhancement at baseline (blue curve), on D8 (red curve) and on D15 (green curve). It was possible to observe an increase in the maximum enhancement and higher area under the enhancement curve early after treatment. **f** The metastatic neck lymph node lesion disappeared after the completion of CCRT and famitinib treatment (arrow), but the patients exhibited thoracic vertebrae metastasis (**g**, arrow) 5 months after complete treatment

**Fig. 6:**
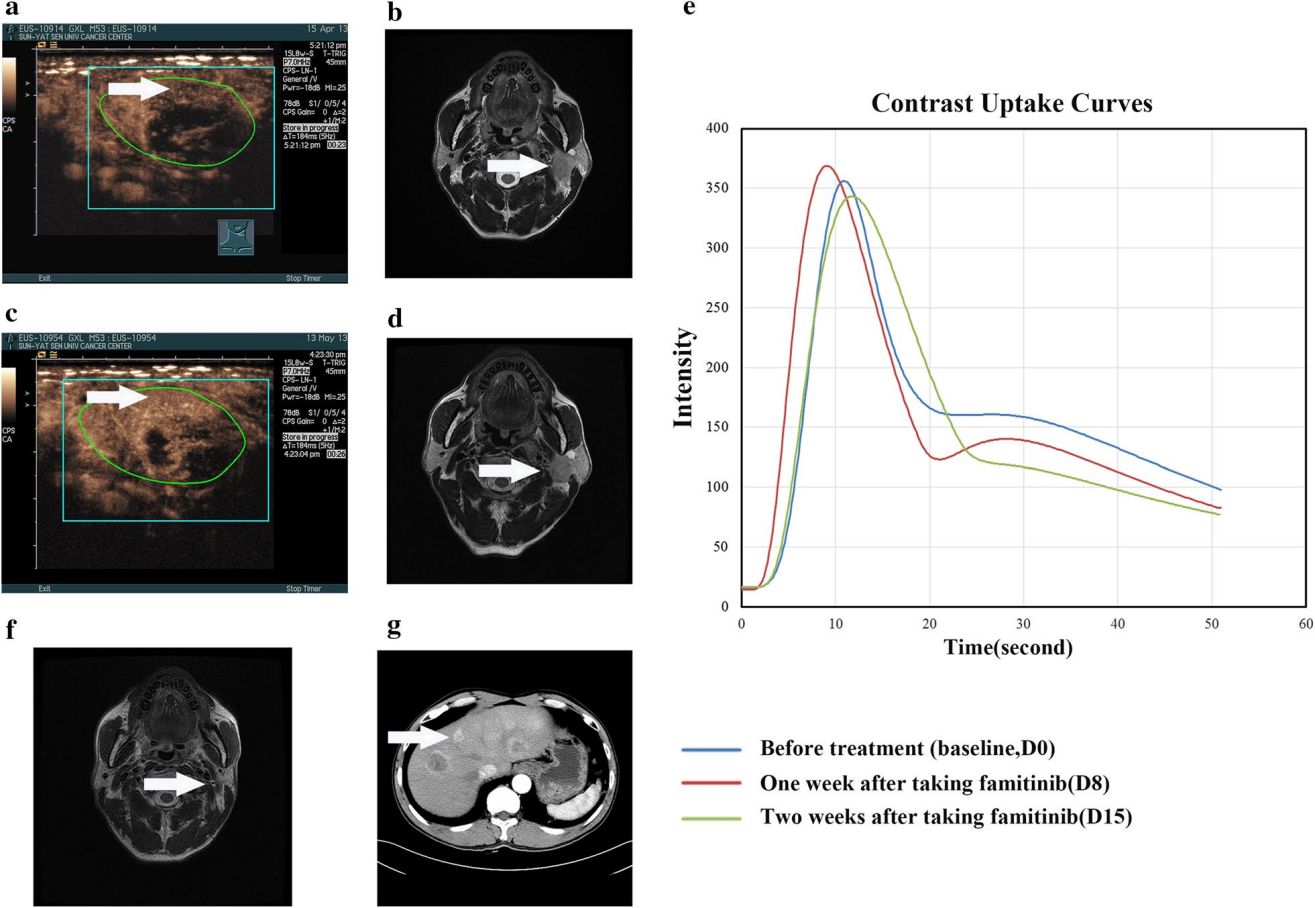
Target neck lymph node lesion in a 54-year-old man (staged with T3N3M0) treated with famitinib (12.5 mg) and CCRT: clinical example of stable disease (SD) according to RECIST (version 1.1.). **a** Contrast-enhanced ultrasound with a strong vascularized lesion (arrow) and corresponding time intensity curve at baseline. **b** The metastatic neck lymph node lesion was evident in the axial T2-weighted MRI image at baseline (arrow). **c** Fourteen days after the onset of famitinib alone treatment, D-CEUS revealed an increase in tumour vascularity density with a slight change in the tumour perfusion parameters, as shown by contrast enhancement pattern and corresponding time-intensity curves. **d** The longest diameter of the metastatic neck lymph node lesion did not change in the axial T2-weighted MRI images at D15 (arrow). **e** Time-intensity curves of tumour enhancement at baseline (blue curve), on D8 (red curve) and on day 15 (green curve). It was possible to observe an increased maximum enhancement and higher area under the enhancement curve at D8 early after treatment. **f** The metastatic neck lymph node lesion disappeared after the completion of CCRT and famitinib treatment (arrow), but the patients exhibited liver metastasis (**g**, arrow) at 11 months after complete treatment

The percentage changes in dynamic functional parameters stratified by progression are shown in Additional file 1: Table S3. The percentage changes in PI, AUC, PW and WIPI at day 0, 8 and 15 were significantly different between patients with and without progression. Furthermore, patients with a perfusion parameter response of less than 30% after taking famitinib for 1 week had a high risk of disease progression (Table 3 and Additional file 1: Fig. S4), suggesting that patients with disease progression had smaller percentage changes in perfusion parameters and were not sensitive to famitinib. Typical clinical examples of the corresponding contrast uptake time-intensity curves for patients with progression at each time point after treatment are shown in Figs. 5 and 6.

**Table 3:** Correlation between D-CEUS parameters and PFS and DMFS

Parameter changes	< 30%	≥ 30%	*P* value
	3-year estimate (%, 95% CI)		
PFS (day 8)			
Peak intensity	90.9 (73.7–100)	44.4 (11.9–76.9)	0.021
Area under the curve	90.0 (71.4–100)	50.0 (31.0–81.0)	0.048
Slope of wash-in (coefficient)	90.9 (73.7–100)	44.4 (11.9–76.9)	0.021
Wash-in perfusion index	90.9 (73.7–100)	44.4 (11.9–76.9)	0.021
DMFS (day 8)			
Peak intensity	90.9 (73.7–100)	55.6 (23.1–88.1)	0.065
Area under the curve	90.0 (71.4–100)	60.0 (23.1–88.2)	0.119
Slope of wash-in (coefficient)	90.9 (73.7–100)	55.6 (23.1–88.1)	0.065
Wash-in perfusion index	90.9 (73.7–100)	55.6 (23.1–88.1)	0.065
PFS (day 15)			
Peak intensity	94.1 (82.9–100)	0.0	< 0.001
Area under the curve	92.3 (77.8–100)	42.9 (6.2–79.6)	0.038
Slope of wash-in (coefficient)	86.7 (69.5–100)	20.0 (0–55.1)	0.002
Wash-in perfusion index	80.0 (59.8–100)	40.0 (0–82.9)	0.072
DMFS (day 15)			
Peak intensity	88.2 (72.9–100)	0.0	< 0.001
Area under the curve	84.6 (65.0–100)	57.1 (20.4–93.8)	0.16
Slope of wash-in (coefficient)	93.3 (80.8–100)	20.0 (0–55.1)	< 0.001
Wash-in perfusion index	86.7 (69.5–100)	21.9 (0–82.9)	0.024

*D*-*CEUS* dynamic contrast enhanced ultrasound, *PFS* progression-free survival, *DMFS* Distant metastasis-free survival, *CI* confidence interval

## Discussion

Findings from this phase I trial of 20 patients showed that the addition of famitinib to chemoradiation has an encouraging tolerability and anticancer profile for patients with NPC. Based on the assessment of safety and efficacy, we recommend famitinib 20 mg combined with chemoradiation (cisplatin 80 mg/m^2^) for phase II testing. Haemorrhage is a well-known complication of sunitinib. However, we recorded no grade 3 or grade 4 haemorrhage when combining famitinib with chemoradiation in this trial. Although two of the first three patients exhibited DLTs when combining famitinib with cisplatin (100 mg/m^2^), less toxicity was observed when cisplatin was reduced to 80 mg/m^2^. Interestingly, we also found that D-CEUS could provide a reliable and early measure of efficacy for NPC patients treated with famitinib.

With the combination with famitinib, 75% of patients received three cycles of concurrent cisplatin, which showed slightly higher rates of compliance with cisplatin during radiation compared with those recorded in the Intergroup 0099 trial (63%) [41], Singapore trial (71%) [42], and Hong Kong NPC-9901 trial (52%) [9]. The 3-year PFS and DMFS were 70% and 75% for these local advanced NPC patients. At the single famitinib stage, most common famitinib-related toxicities were grade I–II, and fewer side effects were noted in this study in terms of leukopenia, neutropenia, thrombocytopenia, and hypertension compared with previously published incidence rates of advanced solid malignancy refractory to standard therapy [28, 29]. This is likely because the patients enrolled in this study had not received any previous treatment, and were in better general health. A meta-analysis of VEGFR tyrosine kinase inhibitors in 23 trials showed that the incidence of bleeding events was 16.7% [43]. Nevertheless, the incidence of haemorrhage in our study was only 10%, which was much lower than the results of Hui et al., who reported high incidence rates of haemorrhage (64.3%) for recurrent or metastatic NPC patients [27]. The incidence of hypertension in this trial was 50%, which was similar to the incidence of hypertension (42.9%) for sunitinib administered to recurrent or metastatic NPC patients [27] and was significantly less than that for sunitinib (92%) in renal cell carcinoma [44]. The most common grade 3–4 adverse events were leukopenia (90%), neutropenia (55%), radiation mucositis (20%), and thrombopenia (20%). The rates of grade 3–4 toxicity of the bone marrow in this trial were higher than in other trials during CCRT in patients with NPC, which were recorded as 12.6%–32% [8, 9] for leukopenia and 13.2% [42] for neutropenia. We considered that famitinib plus CCRT increased the toxicities of the bone marrow, which, however, were tolerable. Grade 3–4 radiation mucositis was found in 20%, which compares favourably to the rates recorded in the Hong Kong NPC-9901 trial (62%) [9] and the Singapore trial (48.1%) [42] as well as with the addition of cetuximab or bevacizumab to standard chemoradiation (77%–87%) [19, 45].

D-CEUS tests found that more than 60% of patients achieved a perfusion parameter response after 2 weeks taking famitinib alone. Previous data have shown the potential of D-CEUS in monitoring the response of anti-angiogenetic agents, and initial contrast uptake was a predictive factor of response to sorafenib and pazopanib in recurrent/metastatic NPC [46, 47]. To our knowledge, this is the first clinical trial to evaluate tumour response to famitinib combined with chemoradiation through D-CEUS for locally advanced NPC. In several patients, famitinib-treated tumours underwent central necrosis or decreases in tumour vascularity, as evidenced by D-CEUS measurements, indicating that famitinib is effective in decreasing tumour vascularity and inducing tumour necrosis before a reduction in tumour volume. In particular, among the parameters evaluated, PI, AUC, PW and WIPI showed an evident reduction early after the onset of famitinib treatment in most of the patients who were free of disease long-term. Patients whose total blood volume described by functional parameters decreased at least 30% after 1 week of famitinib treatment had a higher PFS than those with an increase or a reduction of less than 30%. The same results were obtained when we considered DMFS. Once again, these findings suggest that D-CEUS could be a useful complement to standard anatomic imaging for monitoring early, even long-term, therapeutic effect of famitinib in patients with NPC.

Finally, we should emphasize several limitations of our study. First, this trial is a typical nonrandomized open-label phase I study, and efficacy was only a secondary endpoint. Since many patients were administered at considerably lower doses than the eventual MTD, all efficacy data should be interpreted with caution. Second, the small number of patients decreased the statistical power of our observations. We need phase II study to expand the sample size to further confirm our results. Indeed, variability in measurements is an issue within D-CEUS measurements, even if we found good agreement in a subset of patients. General predictions of the therapeutic effects by perfusion parameters of D-CEUS must be interpreted with caution. From another point of view, the residual cervical lymph nodes in the CCRT and IMRT era are very rare. The clinical applicability of D-CEUS in routine NPC management may be limited. Future work should focus on the development of practical and widely accepted measurements for the calculation of necrosis and for the classification of tumour response based on D-CEUS findings.

## Conclusions

Combined use of famitinib and CCRT (cisplatin, 80 mg/m^2^) is well tolerated at 20 mg/day or lower in patients with NPC. The results also suggest that D-CEUS could be used to evaluate tumour vascularization and efficacy in patients with NPC treated with famitinib.

## Additional file

**Additional file 1: Table S1.** Incidence of late toxicities in the combination group during follow up. **Table S2.** Actual delivered treatments for all enrolled patients. **Table S3.** Percentage changes from baseline of D-CEUS functional parameters stratified by progression after three years of follow up. **Figure S1.** Biomarker expression in NPC tumour tissue and normal nasopharyngeal epithelial cells. **A** VEGFR2; **B** PDGFR; **C** C-kit. VEGFR2, vascular endothelial growth factor receptor; PDGFR, platelet-derived growth factor receptor. **Figure S2.** Serum VEGF (**A**), PDGF (**B**) and SCF (**C**) concentration at baseline, two weeks after taking famitinib, and 12 weeks post-treatment (by ELISA), respectively. VEGF, vascular endothelial growth factor; PDGF, platelet-derived growth factor; SCF, stem cell factor. **Figure S3.** **A** and **B** show the results of longitudinal monitoring of the change in plasma EBV DNA concentrations of 14 patients in continuous remission and 6 patients who exhibited relapse, respectively. **Figure S4.** Progression-free survival (**A**) and distant metastasis-free survival (**B**) in patients with nasopharyngeal cancer treated with intensity-modulated radiotherapy, chemotherapy, and famitinib. Kaplan-Meier survival distributions according to the percentage variation in functional parameters (PI, AUC, PW, and PIWI) at day 8 for famitinib treatment alone. The curves show an association between an early decrease in functional parameters of PI, AUC, PW, and PIWI (after seven days of treatment, D8) and the disease progression. Patients were divided into two groups: those with a percentage decrease in PI (**C**), AUC (**D**), PW (**E**), and PIWI (**F**) greater than or equal to 30% (blue curve) and those with an increase or a percentage decrease lower than 30% (green curve). PI, peak intensity; AUC, area under the time-intensity curve; PW, slope coefficient of wash-in; WIPI, wash-in perfusion index.
