# Supplementary material for: Famitinib in combination with concurrent chemoradiotherapy in patients with locoregionally advanced nasopharyngeal carcinoma: a phase 1, open-label, dose-escalation Study
Source: Cancer Commun (Lond). 2018 Nov 1;38:66. doi: 10.1186/s40880-018-0330-z (PMC6235389; doi:10.1186/s40880-018-0330-z)
Supplement: Supplementary file 1 — Additional file 1: Table S1. Incidence of late toxicities in the combination group during follow up. Table S2. Actual delivered treatments for all enrolled patients. Table S3. Percentage changes from baseline of D-CEUS functional parameters stratified by progression after three years of follow up. Figure S1. Biomarker expression in NPC tumour tissue and normal nasopharyngeal epithelial cells. A VEGFR2; B PDGFR; C C-kit. VEGFR2, vascular endothelial growth factor receptor; PDGFR, platelet-derived growth factor receptor. Figure S2. Serum VEGF (A), PDGF (B) and SCF (C) concentration at baseline, two weeks after taking famitinib, and 12 weeks post-treatment (by ELISA), respectively. VEGF, vascular endothelial growth factor; PDGF, platelet-derived growth factor; SCF, stem cell factor. Figure S3. A and B show the results of longitudinal monitoring of the change in plasma EBV DNA concentrations of 14 patients in continuous remission and 6 patients who exhibited relapse, respectively. Figure S4. Progression-free survival (A) and distant metastasis-free survival (B) in patients with nasopharyngeal cancer treated with intensity-modulated radiotherapy, chemotherapy, and famitinib. Kaplan-Meier survival distributions according to the percentage variation in functional parameters (PI, AUC, PW, and PIWI) at day 8 for famitinib treatment alone. The curves show an association between an early decrease in functional parameters of PI, AUC, PW, and PIWI (after seven days of treatment, D8) and the disease progression. Patients were divided into two groups: those with a percentage decrease in PI (C), AUC (D), PW (E), and PIWI (F) greater than or equal to 30% (blue curve) and those with an increase or a percentage decrease lower than 30% (green curve). PI, peak intensity; AUC, area under the time-intensity curve; PW, slope coefficient of wash-in; WIPI, wash-in perfusion index. [file 40880_2018_330_MOESM1_ESM.docx]

**Supplementary M**[**aterials**](file:///C:\Program%20Files%20(x86)\Youdao\Dict\7.2.0.0703\resultui\dict\?keyword=materials)

**Supplementary Methods**

***Inclusion Criteria***

- Histologically confirmed nasopharyngeal differentiation or undifferentiation carcinoma, WHO II or III
- Newly diagnosed T3-4N1（exception metastatic uni or bil retropharyngeal lymph nodes N1）M0 or any TN2-3(7th UICC/AJCC) M0 locally advanced nasopharyngeal carcinoma
- 18-65 years of age
- ECOG performance status of 0 or 1
- Life expectancy of more than 6 months
- At least one measurable lesion :MRI scan larger than 10 mm in diameter, malignant lymph nodes larger than 10 mm in short axis
- Female: All subjects who are not surgically sterile or postmenopausal must agree and commit to the use of a reliable method of birth control for the duration of the study and for 6 months after the last dose of test article. Child bearing potential, a negative urine or serum pregnancy test result before initiating famitinib. Male: All subjects who are not surgically sterile or postmenopausal must agree and commit to the use of a reliable method of birth control for the duration of the study and for 6 months after the last dose of test article.
- Signed and dated informed consent. Willingness and ability to comply with scheduled visits, treatment plans, laboratory tests, and other study procedure.
- Adequate renal, hepatic, and bone marrow function

***Exclusion Criteria***

- - Before or at the same time any second malignancies except cured basal cell carcinoma of skin and carcinoma in-situ of uterine cervix
  - Any factors that influence the usage of oral administration
  - Known Spinal Cord compression or diseases of brain or pia mater by CT /MRI Screening
  - Imageology shows that tumor lesion less than 5 mm to great vessels(internal carotid and jugular vein)
  - Hemoglobin< 90g/L, platelets < 100×10^9/L, neutrophils < 2×10^9/L, total bilirubin ≥ 1.25×the upper limit of normal(ULN), ALT\AST ≥ 1.5x ULN), serum creatine＞ 1x ULN, creatinine clearance rate < 60ml/min, Cholesterol ＞ 7.75 mmol/L and triglyceride ＞ 3 mmol/L, LVEF: < LLN
  - Hypertensive( more than 140/90 mmHg ), more than class I (NCI CTCAE 3.0 ) myocardial ischemia, arrhythmia(including QTcF:male ≥ 450 ms, female ≥470 ms), or cardiac insufficiency
  - URT: urine protein ≥ ++ and ＞ 1.0 g of 24 h
  - Long-term untreated wounds or fractures
  - PT, APTT, TT, Fbg abnormal, having hemorrhagic tendency (eg. active peptic ulcer disease) or receiving the therapy of thrombolysis or anticoagulation
  - Before the first treatment occurs artery / venous thromboembolic events, such as cerebral vascular accident (including transient ischemic attack), deep vein thrombosis and pulmonary embolism, etc
  - Preexisting thyroid dysfunction, even using medical therapy, thyroid function cannot maintain in the normal range
  - Abuse of Psychiatric drugs or dysphrenia
  - Subject of Viral hepatitis type B or type C
  - Subject of immunodeficiency: HIV positive, or other acquired immunodeficiency, congenital immunodeficiency, or organ transplantation
  - With drug CYP3A4 inhibitor, inducer, or substrate
  - Evidence of significant medical illness that in the investigator's judgment will substantially increase the risk associated with the subject's participation in and completion of the study**.**

***The definition of Planning target volumes (PTVs), high-risk clinical target volume (CTV-1) and low-risk clinical target volume (CTV-2)***

Planning target volumes (PTVs) were expansions of the respective CTVs with a margin of 3 mm, except those close to critical structures, where smaller margins were acceptable. The PTV of the GTVnx was treated with 70 Gy in 33 fractions. The PTVs of CTV-1 and CTV-2 would have received 60 Gy and 54 Gy in 33 fractions, respectively, and 66 to 68 Gy to the PTV of the nodal gross tumour volume (GTVnd). High-risk clinical target volume(CTV-1) was defined as the nasopharynx gross tumour volume plus a 5–10 mm margin (2–3 mm posteriorly if adjacent to the brainstem or spinal cord) to encompass the high-risk sites of microscopic extension and the whole nasopharynx. Low-risk clinical target volume (CTV-2) was defined as the high-risk clinical target volume plus a 5–10 mm margin (2–3 mm posteriorly if adjacent to the brainstem or spinal cord) to encompass the low-risk sites of microscopic extension, including skull base, clivus, sphenoid sinus, parapharyngeal space, pterygoid fossae, posterior parts of the nasal cavity, pterygopalatine fossae, retropharyngeal nodal regions, and the elective neck area from level IB to V.

***Dose Modifications***

According to the CTCAE, v. 4.0, patients will be examined and graded for subjective/objective evidence of developing toxicity each day. In the judgment of the clinician, treatment interruptions are allowed if there is symptomatic mucositis or skin reaction that warrants a break. The treatment is completed as per protocol for treatment breaks up to 14 days. If the break exceeds 14 days, the patient will be removed from protocol treatment. The patient will then complete treatment at the discretion of his/her physician but will be followed and included in the analysis.

If two or more of six patients had a dose-limiting toxicity at the 12.5 mg initial dose level, considering that he maximum tolerated dose of famitinib was 25 mg according to the results of a previous study and that the combination of famitinib with concurrent cisplatin might increase the toxicity, there was no continued dose reduction for famitinib, and the concurrent cisplatin dose level of the first cycle for the remaining enrolled patients was permanently reduced to 80 mg/m². 

Concurrent chemotherapy dosage modifications are based upon nadir counts and interim non-hematologic toxicities of the preceding cycle for cycles 2-3. The dose modifications for chemotherapy (below) are intended to be permanent (i.e., if the patient’s dose is reduced to dose level 1, and it remains at the reduced dose level).

Dose modifications for adverse events during concurrent chemotherapy if the concurrent cisplatin dose in the first cycle was 100 mg/m²

| CisplatinDoseLevels | | |
| --- | --- | --- |
| -2 | -1 | StartingDose |
| 60mg/m2 | 80mg/m2 | 100mg/m2 |

Dose modifications for adverse events during concurrent chemotherapy if the concurrent cisplatin dose in the first cycle was 80 mg/m²

| CisplatinDoseLevels | | |
| --- | --- | --- |
| -2 | -1 | StartingDose |
| 60mg/m2 | 70mg/m2 | 80mg/m2 |

Chemotherapy was not administered until the ANC ≥1.5×10^9^/L and platelets ≥100 nd^9^/L. If these parameters were not met, it was delayed by one week. If the patient still had not recovered to these parameters in one week, cisplatin was discontinued.

If the patient is already at dose level 1, then decrease to dose level 2. If the patient is already at level 2, then discontinue concurrent cisplatin. The third dose of cisplatin should be administered within 1 weeks of the end of radiation. If it cannot be administered in this timeframe, the dose should be stop.

1. Dose adjustment of concurrent cisplatin for hematologic adverse events is based on the nadir counts as follows:

| Neutrophil |  | Platelet | Dose Adjustment |
| --- | --- | --- | --- |
| > 1.0×10^9^/L | and | >75×10^9^/L | Full dose |
| 0.5–<1.0×10^9^/L | and/or | 50–< 75×10^9^/L | Decrease 1 level |
| < 0.5×10^9^/L  or febrileneutropenia  or neutropenicinfection | and/or | 25–< 50×10^9^/L | Decrease 2 levels |

Note: Prophylactic granulocyte colony-stimulating factor was only allowed if a patient had neutropenic infection, febrile neutropenia, or grade 4 neutropenia persisting for 5 days or more on the preceding cycle.

# Dose modifications for nonhematologic adverse events during concurrent chemotherapy

- Renal toxicity

Chemotherapy must be withheld until creatinine clearance > 60 ml/min.

Chemotherapy must be stopped if creatinine clearance < 40 ml/min.

The dosage adjustment for creatinine clearance are as follows:

| Creatinine clearance | Cisplatin |
| --- | --- |
| 50-60 ml / min | Decrease 1 level |
| 40–< 50 ml / min | Decrease 2 level |
| < 40 ml / min | Hold chemotherapy |

***Imaging protocol and data analysis of Contrast-enhanced ultrasound***

Ultrasound imaging was performed at D0 and D8 and d15 after famitinib was administered before starting concurrent chemoradiotherapy. Ultrasound imaging were obtained using an Acuson Sequoia 512 (Siemens, Mountain View, USA) ultrasound unit associated with a 15L8 linear array transducer (7.0～14.0MHz). All ultrasound examinations were performed by two investigator (F Han and JW Wang), who was blinded to the treatment information and clinical outcome. Contrast pulse sequence imaging mode was used for evaluation of tumor (CPS) perfusion (mechanical index: 0.25, frame rate: 5Hz, dynamic range: 78dB, depth: 4 cm). Settings were adjusted at the beginning and maintained constant during all of the experiments.

In this study, SonoVue (Bracco, Milan, Italy) as a second generation contrast agent was used for contrast-enhanced ultrasound imaging. It is a perfluoro gas containing agent with a phospholipids membrane. SonoVue (3.0ml) was injected as a bolus through the retroorbital vein with a 27-gauge needle. In order to decrease the random error, the injection job was performed by the same investigator. Dynamic observations of the longest plane were stored on cine clips up to 60 seconds after the contrast agent injection.

The greatest longitudinal, transverse, and anteroposterior dimensions of each target lymph node were measured before performing contrast enhanced ultrasound imaging. All datasets were transferred to a commercially available workstation （SonoTumor software, Bracco, Milan, Italy）. All the image information was assessed by the same investigator who was blind to the treatment information. A region of interest (ROI) was drawn along the perimeter of each tumor. The region of interest contained necrotic areas, but excluded large vessels. In each ROI, the mean video intensity induced by contrast uptake was calculated for each image and was expressed in decibel. The time-intensity curve for each imaging protocol was plotted, and a mathematic equation model was used to fit the contrast uptake time-intensity curve. Perfusion parameters, including peak intensity (PI), area under the curve (AUC), time to PI (TP), mean transit time (MTT), Slope of wash-in (PW) and wash-in perfusion index (WIPI), were calculated. Peak intensity was defined as the maximum intensity of the signal produced by injection of the contrast agent. Area under the curve was defined as the area under the curve from staring enhancement to peak enhancement. Time to PI was defined as the interval from the beginning of enhancement to peak of the fitted curve. Slope of wash-in was defined as the maximum slope between the time of onset of contrast inflow and the time of peak enhancement, wash-in perfusion index was defined as area under the curve of wash-in divided by rise time. Quality of fit was used to test the fit between the raw data and the fitted mathematic model. Mean transit time(lasting time）was the contrast agent circulation time in ROI tissue.

***The method of immunohistochemistry analysis***

The NPC tissue sections were subjected to citrate-mediated high-temperature antigenic retrievalafter deparaffinization and rehydration, and the endogenous peroxidase activity was blocked. After blocking the non-specific binding, sections were incubated with anti-VEGFR2 (2479s, 1:600, Cell Signaling, Danvers, MA, USA), anti-PDGFR2 (3169s, 1:100, Cell Signaling) and anti-c-kit (ab32363, 1:800, Abcam, Cambridge, MA, USA) antibodies at 4°C overnight, followed by incubation with biotinylated secondary antibody bound to a streptavidin-horseradish peroxidase complex for 30 minutes at room temperature. The bound antibody was visualized by adding 3,3-diaminobenzidine, and the results of immunostaining were independently scored by two well-experienced pathologists blinded to patient data. Tumor cells were defined as positive for VEGFR2, PDGFR2 and C-Kit cytoplasmic staining when ≥5% of the tumor cells presented weak, moderate or strong expression.

***The method of serum biomarker test***

Blood was collected 3ml at baseline (D0), two weeks (D15) after taking famitinib, and 12 weeks (three months). After centrifugation, serum samples were stored at -80°C until final analyses were performed. The serum factors were determined by commercial sandwich ELISA kits (Quantikine, R&D Systems, Minneapolis, MN, USA): VEGF (DVE00), PDGF (DBB00), and SCF (DCK00). All experiments were performed in duplicate, and the results were obtained using a standard curve prepared according to the manufacturer’s instructions.

**Supplementary Tables**

**Table S1. Incidence of late toxicities in the combination group during follow up**

|  | Grade 1-2 | Grade 3 | Grade 4 | Grade 5 | Total |
| --- | --- | --- | --- | --- | --- |
| Hearing impairment | 11(50%) | 1(5%) | 0 | 0 | 11(55%) |
| Skin fibrosis | 10(50%) | 0 | 0 | 0 | 10(50%) |
| Xerostomia | 8(40%) | 0 | 0 | 0 | 8(40%) |
| Peripheral neuritis | 5(25%) | 0 | 0 | 0 | 5(25%) |
| Trismus | 2(10%) | 0 | 0 | 0 | 2(10%) |
| Temporal lobe injury | 1(5%) | 0 | 0 | 0 | 1(5%) |
| Hypothyroidism | 1(5%) | 0 | 0 | 0 | 1(5%) |
| Cranial nerve palsies | 1(5%) | 0 | 0 | 0 | 1(5%) |

**Table S2 Actual delivered treatments for all enrolled patients**

| Treatments | N (%) |
| --- | --- |
| Initial dose of CCRT (100 mg/m^2^,every 3 weeks, n=3) | |
| Dose reduction of concurrent cisplatin |  |
| No dose reduction | 1(33.3%) |
| Less than/equal to 80 mg/m^2^ | 2 (66.7%) |
| Equal to 60 mg/m^2^ | 2 (66.7%) |
| Concurrent cisplatin cycles |  |
| 2 | 2 (66.7%) |
| 3 | 1(33.3%) |
| Famitinib adherence |  |
| Famitinib delay | 0 |
| Famitinib pause | 2 (66.7%) |
| Initial dose of CCRT (80 mg/m^2^, every 3 weeks, n=17) | |
| Dose reduction of concurrent cisplatin |  |
| No dose reduction | 4 (23.5%) |
| Less than/equal to70 mg/m^2^ | 13 (76.5%) |
| Equal to 60 mg/m^2^ | 11 (64.7%) |
| Concurrent cisplatin cycles |  |
| 2 | 3 (17.6%) |
| 3 | 14(82.4%) |
| Famitinib adherence |  |
| Famitinib delay | 2 (11.8%) |
| Famitinib pause | 2 (11.8%) |
| Total CCRT (n=20) |  |
| 2 | 5 (25.0%) |
| 3 | 15(75.0%) |

Abbreviations: CCRT, concurrent chemoradiotherapy.

**Table S3 Percentage changes from baseline of D-CEUS functional parameters stratified by progression after three years of follow up**

| Parameter | Day 8 (%) | | P value | Day 15 (%) | P value |
| --- | --- | --- | --- | --- | --- |
| Peak intensity | |  | 0.003 |  | 0.032 |
| Progression | | -5.2(-29.2, 1.2) |  | -23.5(-53.6, 33.2) |  |
| Non-progression | | -49.1(-62.7, -26.1) |  | -60.8(-72.2, -43.0) |  |
| Area under the curve | |  | 0.048 |  | 0.039 |
| Progression | | -7.4(-31.6, 20.2) |  | -11.1(-57.7, 27.8) |  |
| Non-progression | | -47.0(-60.9, -13.0) |  | -70.9(-73.6, -31.4) |  |
| Time to peak intensity | |  | 0.099 |  | 0.564 |
| Progression | | -9.3(-17.3, 8.1) |  | -13.7(-18.3, 81.0) |  |
| Non-progression | | 15.9(-5.5, 33.8) |  | 0.3(-2.0, 36.1) |  |
| Mean transit time | |  | 1.000 |  | 0.621 |
| Progression | | -9.4(-26.0, 17.4) |  | 10.9(-49.1, 33.4) |  |
| Non-progression | | -12.6(-33.3, 27.9) |  | -1.1(-32.7, 87.8) |  |
| Slope of wash-in | |  | 0.026 |  | 0.058 |
| Progression | | -9.6(-26.5, 4.4) |  | -30.3(-52.0, 10.2) |  |
| Non-progression | | -55.6(-71.4, -24.0) |  | -69.3(-43.1, -80.0) |  |
| Wash-in perfusion index | |  | 0.026 |  | 0.039 |
| Progression | | -5.4(-17.9, 10.4) |  | -14.0(-66.1, 8.5) |  |
| Non-progression | | -57.1(-70.0, -11.3) |  | -68.6(-36.7, -77.0) |  |

Data are expressed as median percentage change values. Numbers in parentheses are the first and third quartile, respectively. *P* values are for differences between patients with progression or non-progression

**Supplementary Figures**


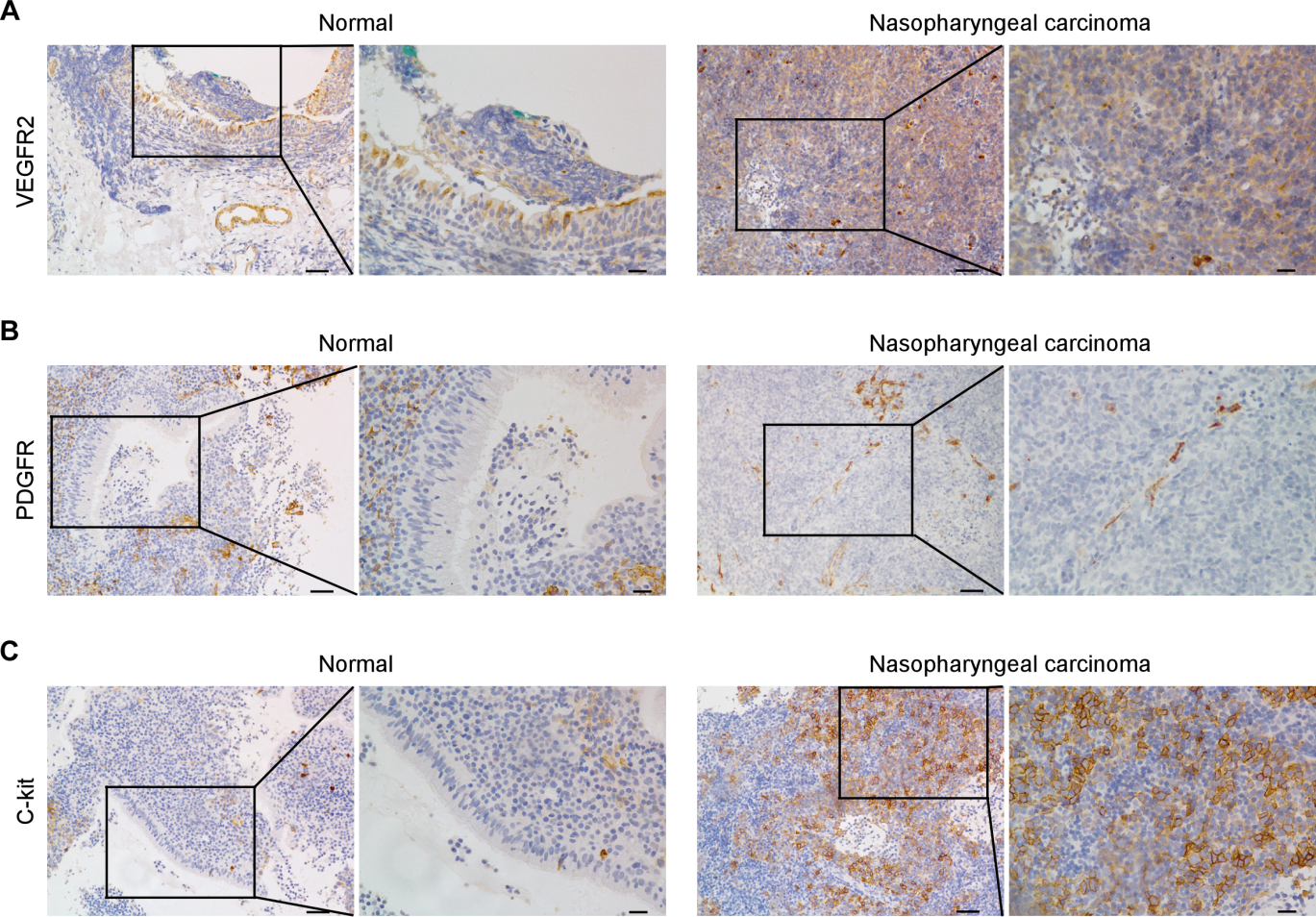


**FigureS1.** Biomarker expression in NPC tumour tissue and normal nasopharyngeal epithelial cells. A, VEGFR2; B, PDGFR; C, C-kit, VEGFR2, vascular endothelial growth factor receptor; PDGFR, platelet-derived growth factor receptor.


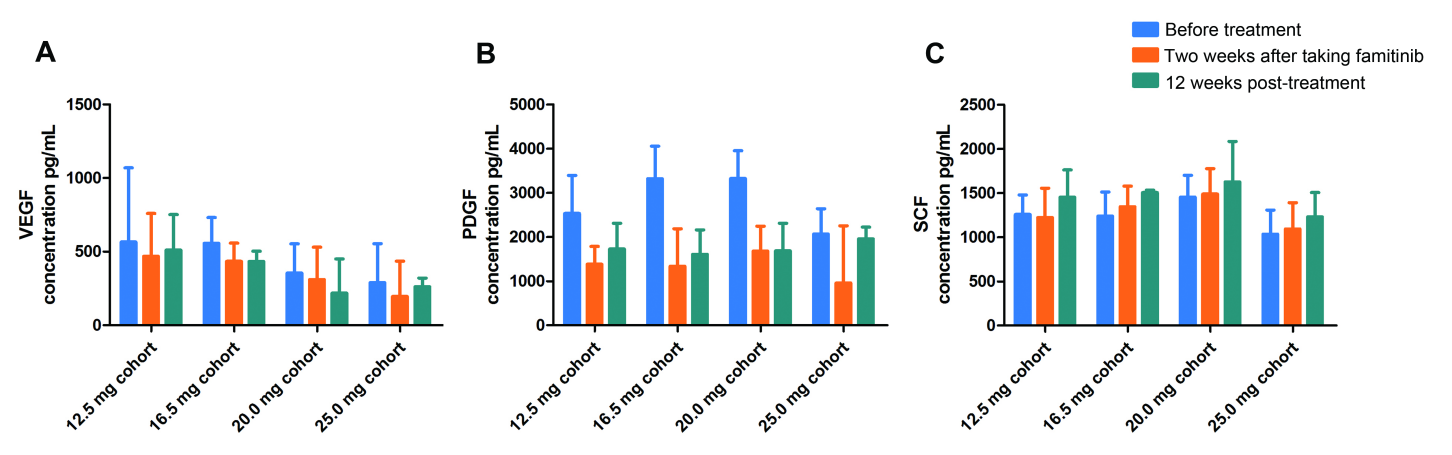


**Figure S2**. Serum VEGF (A), PDGF (B) and SCF (C) concentration at baseline, two weeks after taking famitinib, and 12 weeks post-treatment (by ELISA), respectively. VEGF, vascular endothelial growth factor; PDGF,platelet-derived growth factor; SCF, stem cell factor


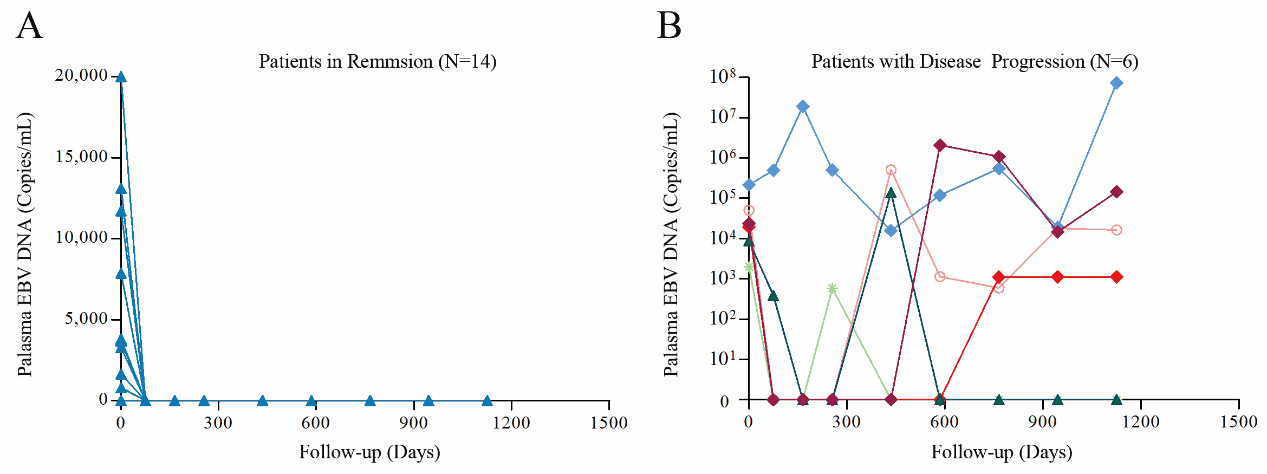


**FigureS3.**  A and B show the results of longitudinal monitoring of the change in plasma EBV DNA concentrations of 14 patients in continuous remission and 6 patients who exhibited relapse, respectively.


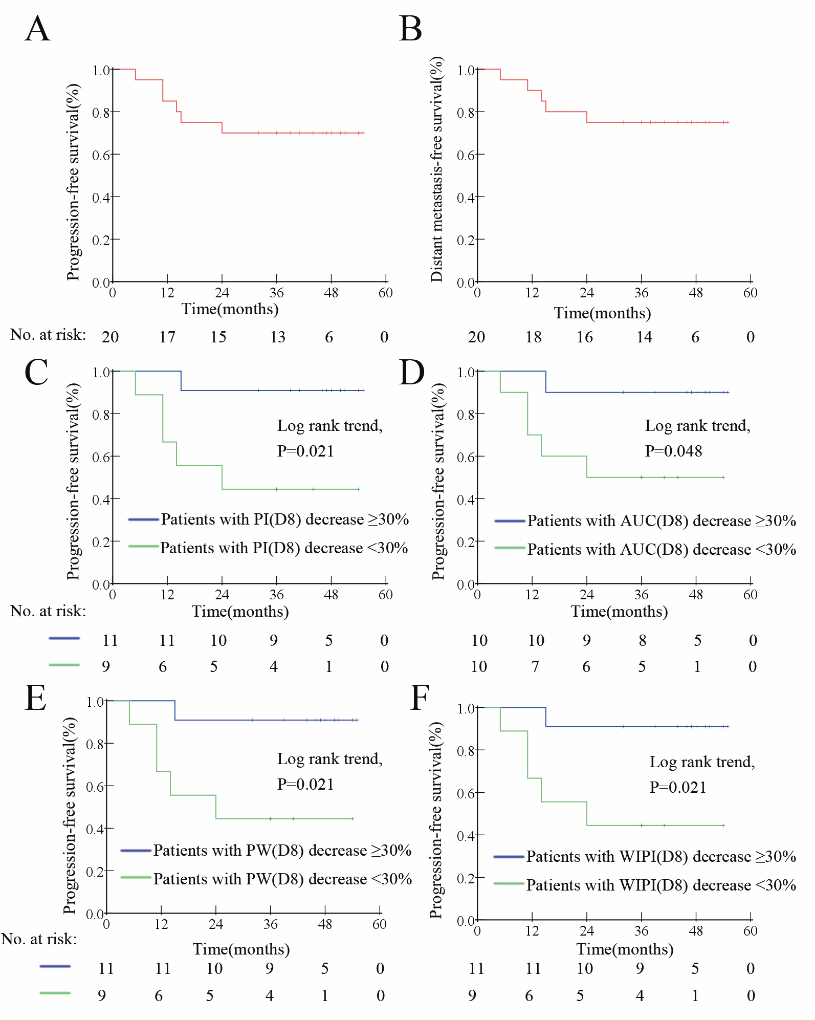


**Figure S4.**  Progression-free survival (A) and distant metastasis-free survival(B) in patients with nasopharyngeal cancer treated with intensity-modulated radiotherapy, chemotherapy, and famitinib. Kaplan-Meier survival distributions according to the percentage variation in functional parameters (PI, AUC, PW, and PIWI) at day 8 for famitinib treatment alone. The curves show an association between an early decrease in functional parameters of PI, AUC, PW, and PIWI (after seven days of treatment, D8) and the disease progression. Patients were divided into two groups: those with a percentage decrease in PI(C), AUC(D), PW(E), and PIWI(F) greater than or equal to 30% (blue curve) and those with an increase or a percentage decrease lower than 30% (green curve). PI, peak intensity; AUC, area under the time-intensity curve; PW, slope coefficient of wash-in; WIPI, wash-in perfusion index.
